# Supplementary material for: Structural mechanism of CB1R binding to peripheral and biased inverse agonists
Source: Nat Commun. 2024 Dec 18;15:10694. doi: 10.1038/s41467-024-54206-0 (PMC11655885; doi:10.1038/s41467-024-54206-0)
Supplement: Supplementary file 1 — Supplementary Information [file 41467_2024_54206_MOESM1_ESM.pdf]

Supplementary Table 1

Cryo-EM data collection and refinement statistics

	Taranabant	MRI-1867	MRI-1891
**Data collection and processing**			
Magnification	81,000	81,000	81,000
Voltage (kV)	300	300	300
Electron exposure (e^{-}/A^{2})	72.2	50.0	60.0
Defocus range (μm)	1.4-2.4	1.4-2.4	1.4-2.4
Pixel size (Å)	1.07	1.08	1.07
Symmetry imposed			
Initial particle images (no.)	6,400,000	1,470,000	1,920,000
Final particle images (no.)	308,624	102,049	99,842
Map resolution (Å)	3.5	3.3	3.15
FSC threshold	0.143	0.143	0.143
Map resolution range (Å)	3.0-5.7	3.0-5.7	3.0-5.7
**Refinement**			
Initial model used (PDB code)	6KQI, 5VNV	9B9Y	9B9Y
Model Resolution (Å)	3.2	3.0	2.7
FSC threshold	0.5	0.5	0.5
Map sharpening B factor (Å)	-60	-70	-60
Model composition			
Non-hydrogen atoms	4753	4745	4742
Protein residues	601	601	601
Ligands	Taranabant	MRI-1867	MRI-1891
B factors (Å)			
Protein	108.24	73.33	54.26
Ligand	112.69	87.68	75.09
R.m.s deviations			
Bond lengths (Å)	0.003	0.004	0.003
Bond angles (°)	0.749	0.781	0.493
Validation			
MolProbity score	1.36	1.40	1.38
Clashscore	3.97	4.51	6.20
Poor rotamers (%)	0	0	0
Ramachandran plot			
Favored (%)	96.98	96.98	97.82
Allowed (%)	3.02	3.02	2.18
Disallowed (%)	0	0	0

Supplementary Figure 1

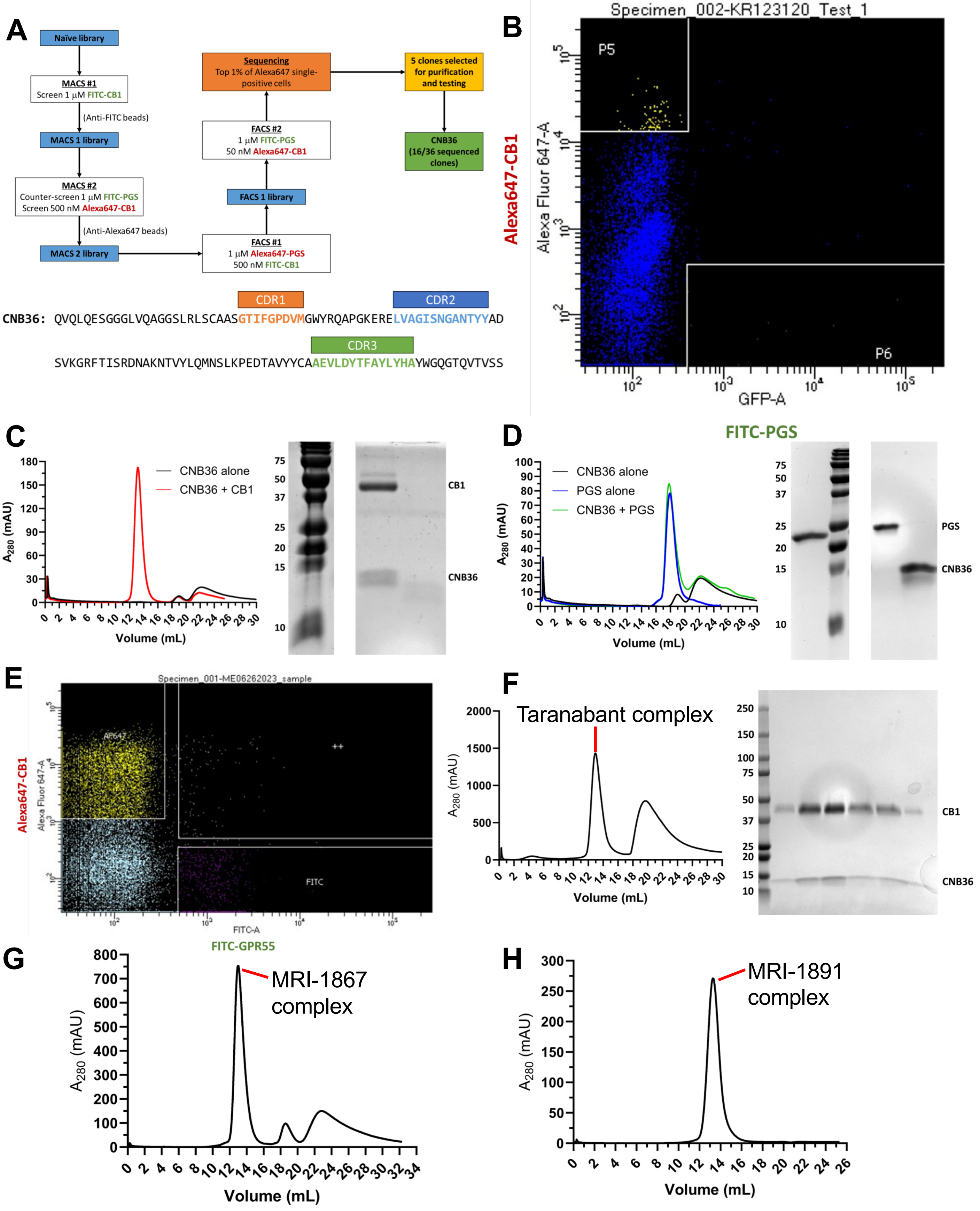

Supplementary Figure 1. (A-H) Selection and biochemical characterization of CNB36

(A) Overall strategy for nanobody selection and sequence of CNB36.

(B) FACS sort of a yeast display nanobody library against Alexa647-labeled CB1-PGS bound to taranabant (Y-axis) versus FITC-labeled PGS alone (X-axis) from the final round of selection that gave rise to CNB36. The indicated gate (P5) contains the highly fluorescent CB1-PGS single positive population, which was then analyzed by sequencing of individual clones.

(C) Binding of CNB36 to CB1-PGS, read out by size exclusion chromatography and SDS-PAGE. Lane #1 contains a fraction from the center of the peak corresponding to CB1-PGS, lane #2 is a loading control for CNB36.

(D) Assessment of binding of CNB36 to PGS alone, as in C.

(E) Analytical flow cytometry comparing binding of CNB36-expressing yeast to Alexa647-labeled CB1-PGS (Y-axis) versus FITC-labeled GPR55-PGS (X-axis).

(F) Final sample used for cryo-EM grid preparation by size excusion chromatography and SDS-PAGE for the CB1-PGS-CNB36 complex bound to taranabant.

(G) Sample for MRI-1867

(H) Sample for MRI-1891

Supplementary Figure 2

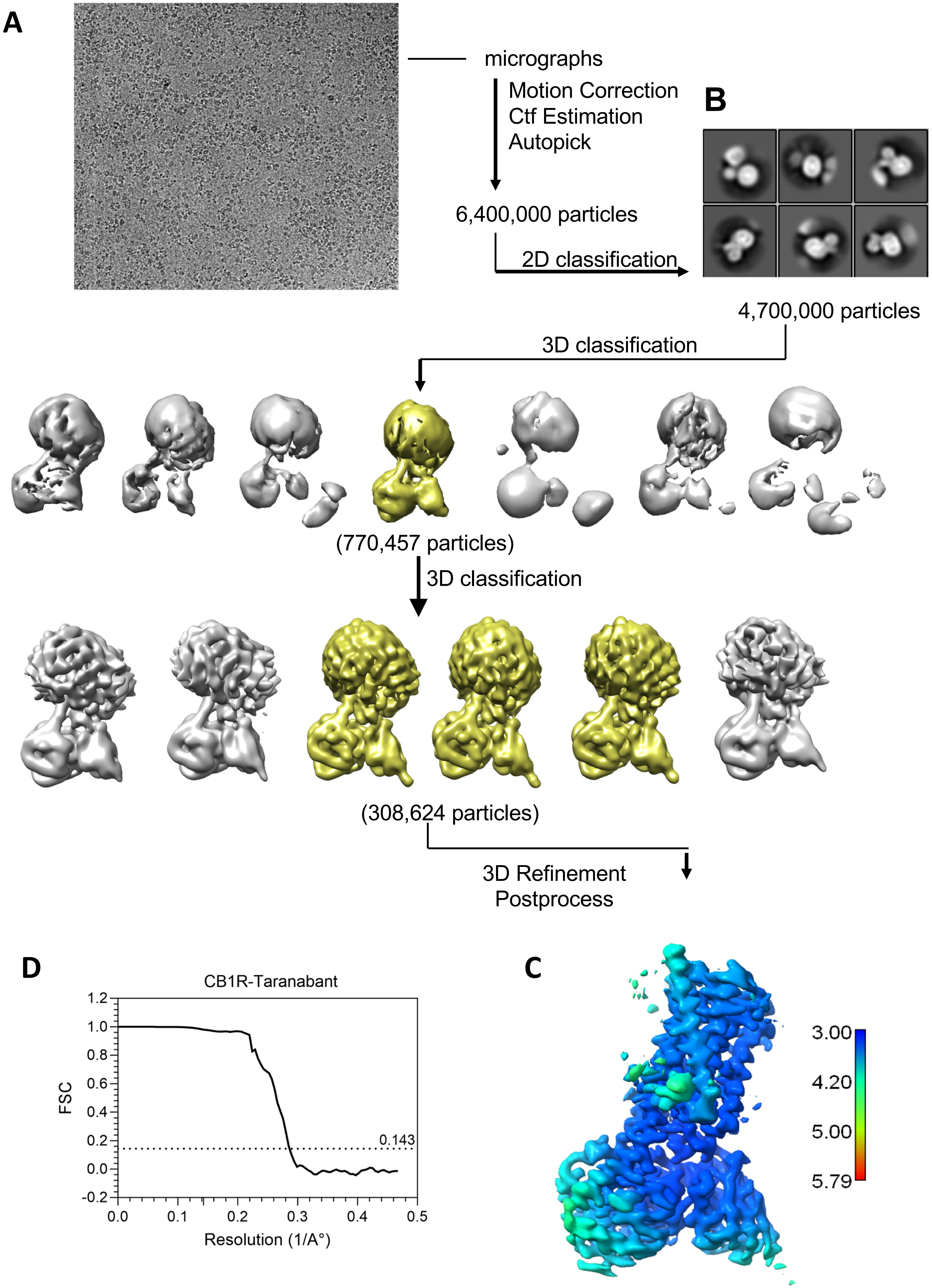

Supplementary Figure 2. (A-D) Cryo-EM data processing of CB_1R-PGS-CNB36 in complex with taranabant

(A) Representative cryo-EM micrograph
(B) Representative 2D class averages and typical workflow demonstrating data processing in RELION 3.1
(C) Local resolution distribution of taranabant bound CB_1R-PGS-CNB36 complex colored according to local resolution estimation in RELION and displayed by ChimeraX 1.3
(D) Fourier shell correlation (FSC) curves comparing two halves of the data from the 3D reconstruction, generated during postprocessing in RELION 3.1. The dashed line indicates FSC 0.143.

Supplementary Figure 3

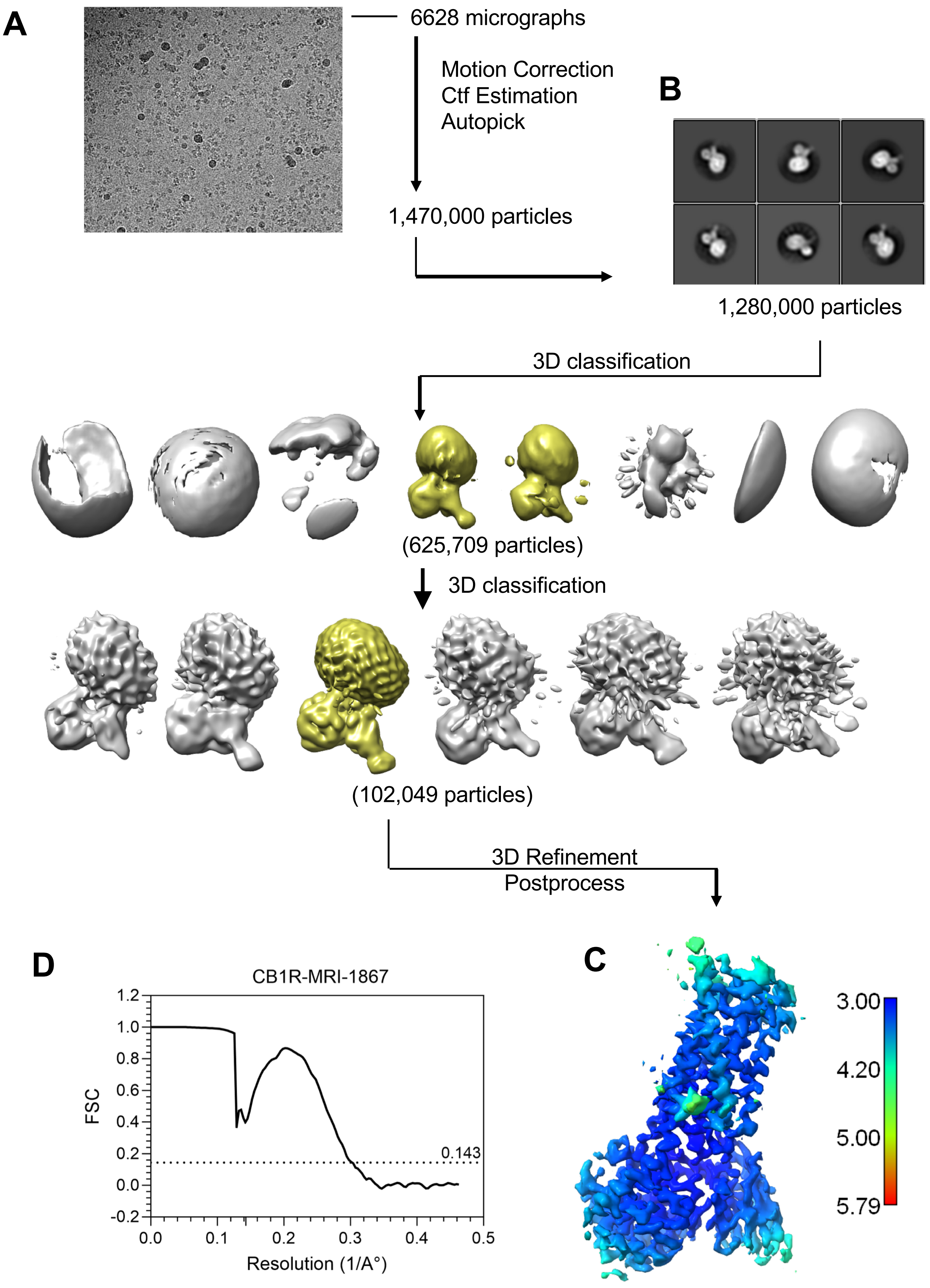

Supplementary Figure 3. (A-D) Cryo-EM data processing of CB_1R-PGS-CNB36 in complex with MRI-1867

(A) Representative cryo-EM micrograph
(B) Representative 2D class averages and typical workflow demonstrating data processing in RELION 3.1
(C) Local resolution distribution of taranabant bound CB_1R-PGS-CNB36 complex colored according to local resolution estimation in RELION and displayed by ChimeraX 1.3
(D) Fourier shell correlation (FSC) curves comparing two halves of the data from the 3D reconstruction, generated during postprocessing in RELION 3.1. The dashed line indicates FSC 0.143.

Supplementary Figure 4

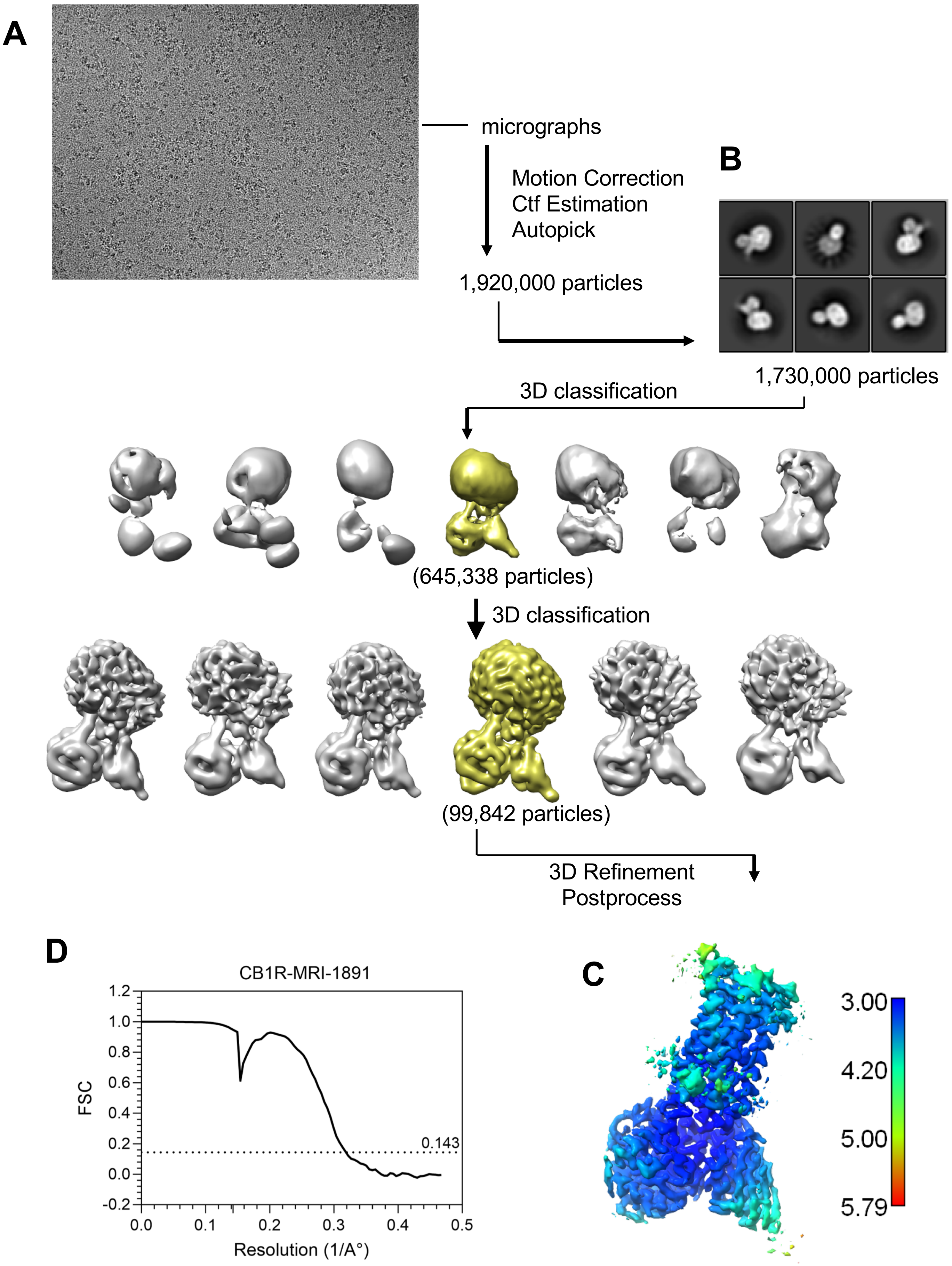

Supplementary Figure 4. (A-D) Cryo-EM data processing of CB_1R-PGS-CNB36 in complex with MRI-1891

(A) Representative cryo-EM micrograph
(B) Representative 2D class averages and typical workflow demonstrating data processing in RELION 3.1
(C) Local resolution distribution of taranabant bound CB_1R-PGS-CNB36 complex colored according to local resolution estimation in RELION and displayed by ChimeraX 1.3
(D) Fourier shell correlation (FSC) curves comparing two halves of the data from the 3D reconstruction, generated during postprocessing in RELION 3.1. The dashed line indicates FSC 0.143.

Supplementary Figure 5

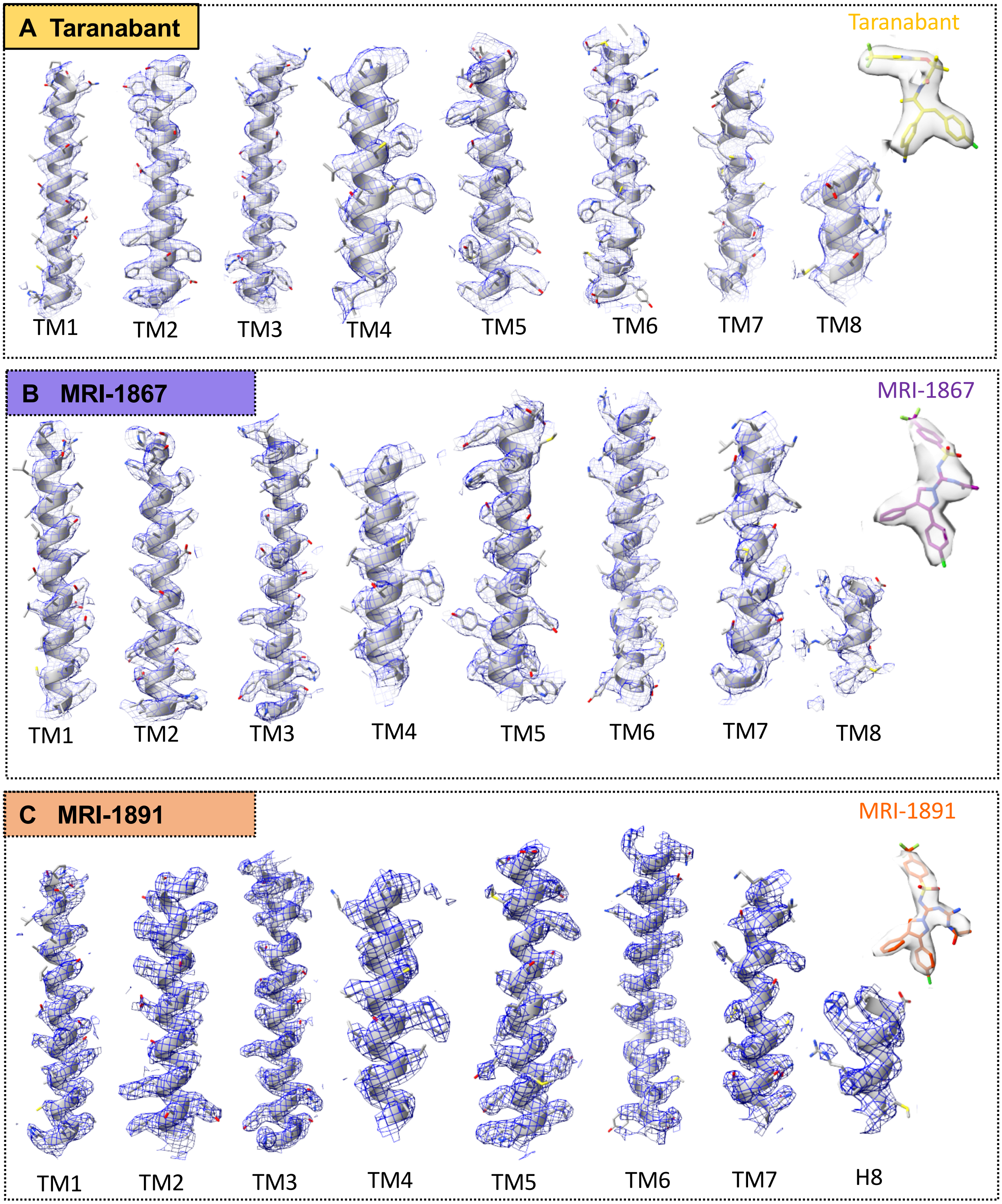

Supplementary Figure 5. Density for FFAR1 in the cryo-EM structures. (A) Transmembrane helix and ligand density in the Taranabant bound CB_1R-CNB36 cryo-EM map. Density map (and model) was displayed by chimeraX 1.3 with contour level 0.0234 (5.2 sigma). **(B)** Transmembrane helix and ligand density in the MRI-1867 bound CB_1R-CNB36 cryo-EM map. Density map (and model) was displayed by chimeraX 1.3 with contour level 0.0147 (3.5 sigma). **(C)** Transmembrane helix and ligand density in the MRI-1891 bound CB_1R-CNB36 cryo-EM map. Density map (and model) was displayed by chimeraX 1.3 with contour level 0.0326 (4.7 sigma).

Supplementary Figure 6

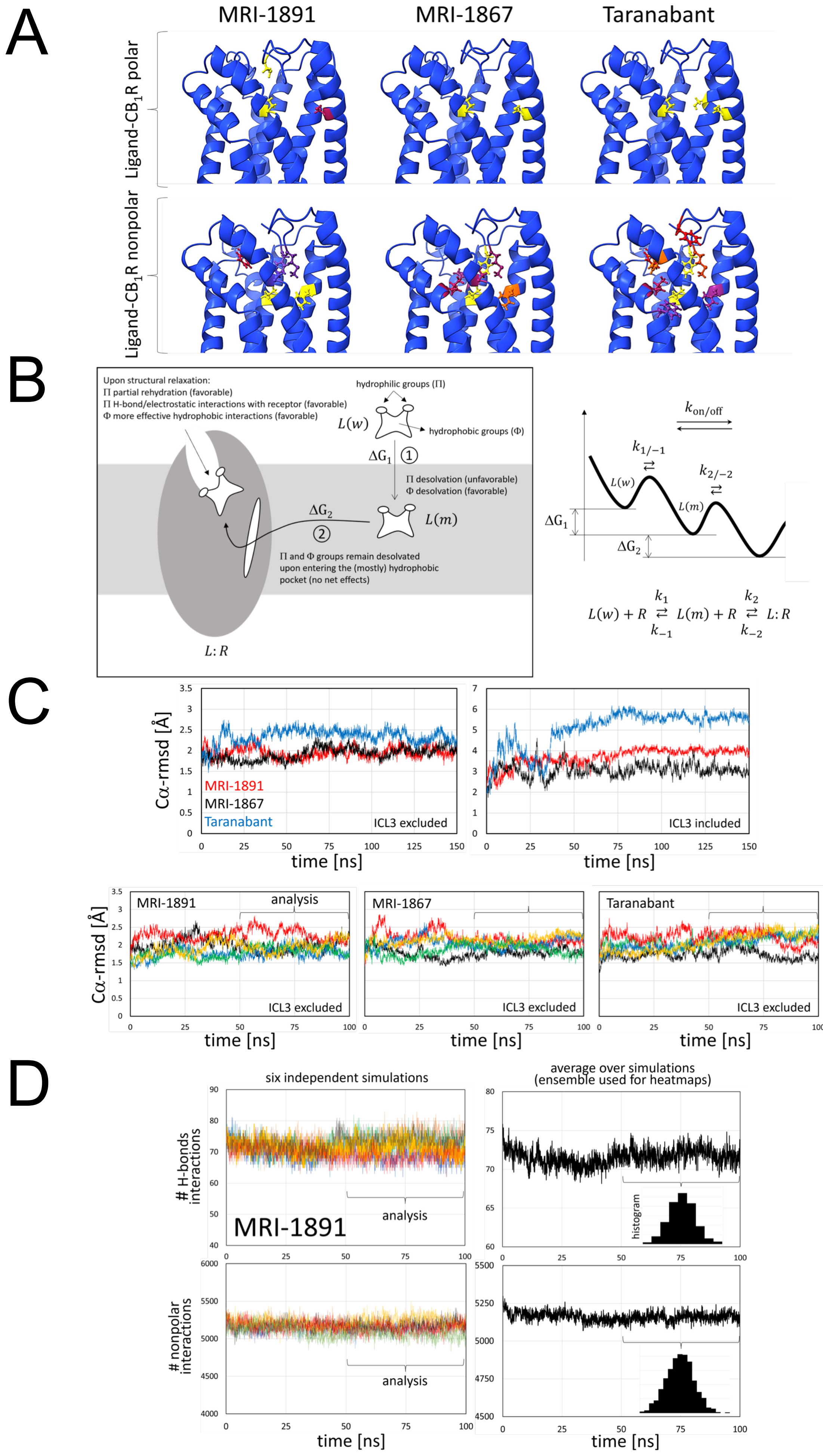

Supplementary Figure 6. Molecular dynamics simulations. **(A)** The most prevalent polar and nonpolar interactions between the ligands and receptors were observed during the dynamics. Heatmaps mapped on the same reference structure according to frequency/strength; yellow: high (> 80%), blue: low (<20%; side chains omitted). See Supp. ChimeraX session. Polar interactions: MRI-1891: C107, S123, S383; MRI-1867: S123, S383; Taranabant: S123, S173, S383. Nonpolar interactions: MRI-1891: F102, M103, F170, V196, F268, S383 (Cb); MRI-1867: F102, M103, F170, L193, L359, S383; Taranabant: F102, M103, F108, F170, V196, F268, W356, L359, F379. **(B)** Proposed mechanism for the high binding affinity of MRI-1891. The balance between hydrophilic and hydrophobic moieties in the ligands determines the partition between water and membrane environments; the physicochemical properties of Arm4, which faces the extracellular opening upon binding, allows for partial compensation of the water-to-membrane desolvation penalty of its polar groups through rehydration and a more favorable packing of its hydrophobic groups. **(C)** Time evolution of the CB_1R Ca-rmsd for the three complexes after thermal equilibration. The reference structures are the initial constructs (cryo-EM with modeled ICL3; cf. Methods). Extended simulation (upper row) with the ICL3 excluded (left panel) and included (right); five additional simulations with the ICL3 excluded (lower row). A quick departure of 1.5-2.5 Å from the initial structure is observed in all cases, with stabilization stabilized after 20-30 ns; similar thermal (< 0.5 Å) and conformational (< 1 Å) fluctuations are observed after ~50 ns. The ICL3 undergoes larger changes and takes longer to stabilize (~50 ns). All the analysis (Fig. 5) was restricted to the 50-100 ns range and omitted ICL3 (cf. Methods and Results). **(D)** Besides the Ca-rmsd, several other metrics were used to assess stabilization and statistical robustness. All the statistics in Fig. 5, including heatmaps and clustering, are obtained after data from the six independent simulations were collected into a single ensemble. The figure shows two metrics for the CB_1R:MRI-1891 complex, typical of the statistics used for comparative analysis with the other complexes.

Supplementary Figure 7

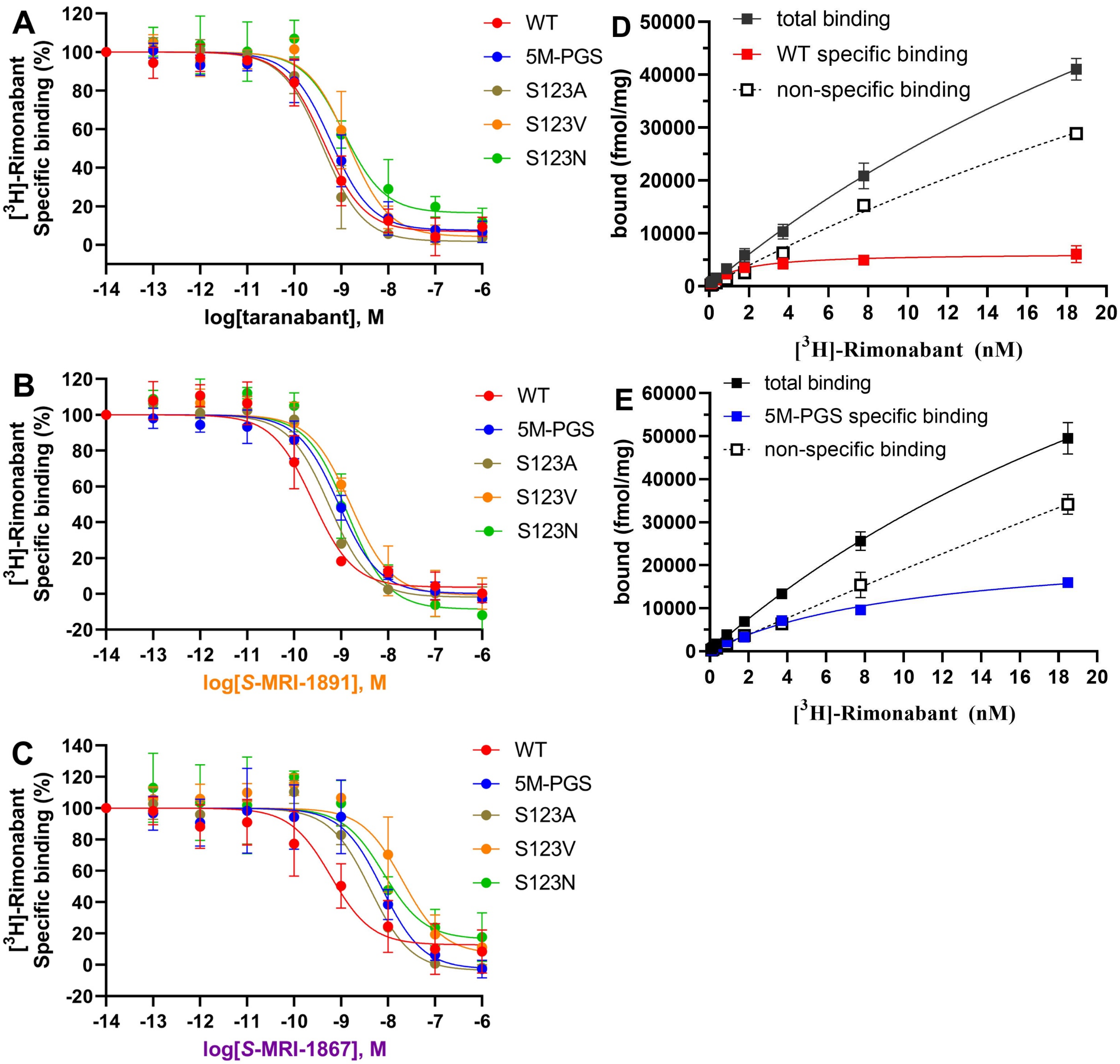

Supplementary Figure 7. Binding of ligands to CB1R membranes. (A) Binding of antagonist taranabant in [^{3}H]-rimonabant competition assays to CB$_1$R-WT, CB$_1$R-5M-PGS, and CB$_1$R mutant cell membrane homogenates. Curves represent the specific binding of the radioligand in percentage in the presence of increasing concentrations (10−13–10−6 M) of the indicated ligands. Values represent mean ± s.e.m from n=6 (WT, 5M, S123N) or n=4 (S123A and S123V) independent experiments, each done in triplicate. Source data are provided with this manuscript as Source Data file. K_i values are shown in Supp. Table 2; (**B**) Analogous assay to panel A, except with antagonist *S*-MRI-1891. Values represent mean ± s.e.m from n=3 (WT), n=6 (5M), or n=4 (S123N, S123A and S123V) independent experiments, each done in triplicate. Source data are provided with this manuscript as Source Data file. K_i values are shown in Supp. Table 2; (**C**) Analogous assay to panel A, except with antagonist *S*-MRI-1867. Values represent mean ± s.e.m from n=6 (WT and 5M) or n=4 (S123N, S123A and S123V) independent experiments, each done in triplicate. Source data are provided with this manuscript as Source Data file. K_i values are shown in Supp. Table 2; **(D)** Saturation binding of the tritiated antagonist [3H]-rimonabant to wild-type CB$_1$R membranes. Values represent mean ± s.e.m from n=4 independent experiments, each done in triplicate. Source data are provided with this manuscript as Source Data file. K_d and B_{max} values are shown in Supp. Table 3; (**E**) Analogous assay to panel D, except for CB$_1$R-5M-PGS membranes. Values represent mean ± s.e.m from n=4 independent experiments, each done in triplicate. Source data are provided with this manuscript as Source Data file. K_d and B_{max} shown in Supp. Table 3.

Supplementary Table 2

Competition binding affinity (K_i) of cannabinoid receptor antagonists on Sf9 cell membranes Error bars represent S.E.M. for three to six separate experiments performed in duplicate.

compounds	Ki ± S.E.M. (nM) vs [^{3}H]-Rimonabant				
	**CB1 WT**	**CB1-5M-PGS**	**S123A**	**S123V**	**S123N**
**Taranabant**	**0.23** ± 0.09	**0.58** ± 0.1	**0.22** ± 0.06	**1.2** ± 0.2	**0.89** ± 0.1
**(S)-MRI-1867**	**0.31** ± 0.08	**7.3** ± 0.4	**2.4** ± 0.2	**13** ± 1.2	**5.3** ± 0.4
**(S)-MRI-1891**	**0.09** ± 0.02	**0.88** ± 0.1	**0.29** ± 0.01	**1.2** ± 0.03	**0.86** ± 0.1

Supplementary Table 3

Saturation binding of [^{3}H]rimonabant to wild-type CB_1R and mutant Sf9 cell membranes. Error bars represent S.E.M. for three to six separate experiments performed in duplicate.

	**CB1 WT**	**CB1-5M-PGS**	**S123A**	**S123V**	**S123N**
**B_{max} ± S.E.M. (fmol/mg)**	**5906 ± 198**	**25716 ± 569**	**30499 ± 647**	**34032 ± 447**	**9009 ± 469**
**K_d ± S.E.M. (nM)**	**1.6 ± 0.3**	**10.7 ± 0.9**	**2.2 ± 0.03**	**11.9 ± 0.4**	**8.6 ± 0.6**
