# Supplementary material for: Structural mechanism of CB1R binding to peripheral and biased inverse agonists
Source: Nat Commun. 2024 Dec 18;15:10694. doi: 10.1038/s41467-024-54206-0 (PMC11655885; doi:10.1038/s41467-024-54206-0)
Supplement: Supplementary file 2 — Description of Additional Supplementary Files [file 41467_2024_54206_MOESM2_ESM.docx]

**Supplementary Dataset 1.** The archive includes a README file detailing its content: scripts (configuration files) for NAMD simulations, topology and parameter files for the all-atom CHARMM force field, initial coordinates of the system components, and snapshots of the three complexes throughout the dynamics. It also includes scripts for batch submission to an HPC cluster with a SLURM scheduler.
